# Can exchange transfusion be replaced by double-LED phototherapy?

**DOI:** 10.1515/med-2021-0320

**Published:** 2021-07-02

**Authors:** Shinya Abe, Kazumichi Fujioka

**Affiliations:** Department of Pediatrics, Kobe University Graduate School of Medicine, 7-5-1 Kusunoki-Cho, Chuo-Ku, Kobe 650-0017, Hyogo, Japan

**Keywords:** neonatal jaundice, bilirubin, irradiance, LED

## Abstract

Phototherapy is a conventional treatment for neonatal jaundice and widely considered as a safe procedure. Recent developments in light-emitting diode (LED) phototherapy devices have made more effective treatments possible. Exchange transfusion (ET) is typically applied for cases of refractory severe hyperbilirubinemia despite its risk of various complications. Since the therapeutic effect of phototherapy is correlated with its irradiance, ET may be avoided by performing phototherapy with higher irradiation. Recently, we adopted double-LED phototherapy as a bridging treatment to ET to treat a case of severe hyperbilirubinemia. In this case, the continual increase of bilirubin levels was suppressed immediately after its administration, and ET was not required. Throughout the treatment, no complications or increase in oxidative stress was observed. In addition, neurodevelopment was appropriate for the patient’s age at the 1-year follow-up, and no findings of kernicterus, including physical and magnetic resonance imaging findings, were observed. We hypothesized that double-LED phototherapy may be a good treatment strategy to replace ET for infants with severe hyperbilirubinemia; however, further investigations regarding safety issues including acute and long-term complications are needed before clinical adaptation.

## 1 Introduction

Phototherapy is a conventional treatment for neonatal jaundice, but exchange transfusion (ET) is necessary for cases of refractory severe hyperbilirubinemia [1]. Although phototherapy is widely considered a safe procedure [2], ET is known to be associated with various complications such as metabolic acidosis, hypotension, and convulsions [3]. Recent studies indicate that the estimated risks of ET-related mortality, serious complications, and even minor complications are 0–3%, 2–12%, and 40–70%, respectively [3,4,5]. In addition, with the development of light-emitting diode (LED) phototherapy, which was shown to cause significantly greater decreases in total serum bilirubin levels and increases in urinary lumirubin levels in comparison with conventional phototherapy [2], the use of ET for the treatment of severe hyperbilirubinemia has been declining [5]. In fact, there have been no ET treatments for neonatal jaundice in our facility in the past 3 years; subsequently, there have been concerns that a decrease in proficiency may lead to an increased risk of ET-related complications.

Meanwhile, the therapeutic effect of phototherapy is affected by the wavelength and intensity of light irradiation, body surface area of the child exposed to light, and irradiation time [6]. Of these factors, the intensity of irradiation can be changed most easily, and the benefits of intensified phototherapy have been previously addressed in 1997 [7]. Hansen reported excellent bilirubin reduction by intensified phototherapy with breastfeeding in patients with severe hyperbilirubinemia who had serum bilirubin levels >30 mg/dL [7]. Furthermore, these rapid and effective jaundice treatments including intensified phototherapy (>30 µW/cm^2^/nm) were subsequently labeled as a “crash-cart approach” by Smitherman and Hansen. [8,9,10].

Therefore, it may be possible to avoid ET for severe hyperbilirubinemia by performing phototherapy with a higher irradiance. Before the commercialization of LED phototherapy, double-fluorescent tube light phototherapy was performed in cases of severe hyperbilirubinemia, in which conventional fluorescent tube phototherapy was not effective, with the aim of improving the irradiance and irradiating area of the skin [11,12]. Although the basic premise is the same, the PT described by Hansen in 1997 was performed by using “old-fashioned” fluorescent bulbs, yielding less irradiance can be achieved by modern PT equipment using LED systems [7].

Since 2017, we have used new treatment criteria for neonatal jaundice proposed by Morioka [1]. The criteria determine the therapeutic indications for low-mode phototherapy (LP, 10–15 µW/cm^2^/nm), high-mode phototherapy (HP, 30 µW/cm^2^/nm), and ET based on the serum total bilirubin and unbound bilirubin levels. Recently, we adopted double-LED phototherapy using two different types of LED phototherapy equipment, which could theoretically apply higher irradiance than HP, as a bridging treatment to ET to treat a case of severe hyperbilirubinemia refractory to single-LED phototherapy.

## 2 Methods

**Figure 1:**
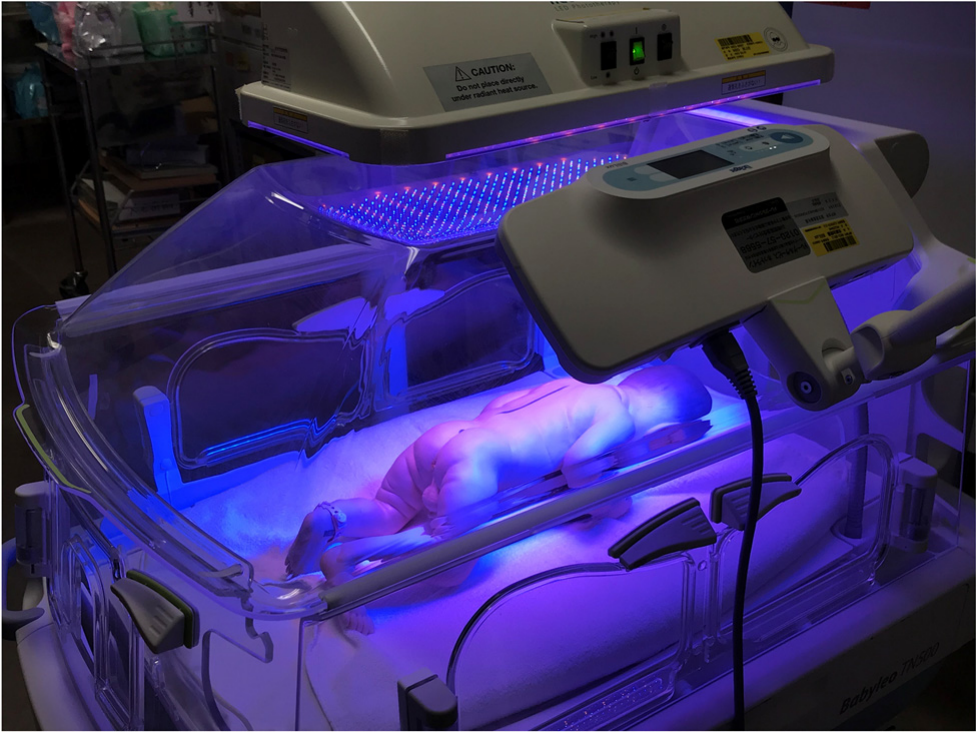
Schematic for double-LED phototherapy. Double-LED phototherapy is performed from above the incubator using neoBLUE and diagonally above the incubator using BiliLux. Before starting treatment, it is important to adjust the device’s position so that the baby is in the center of the irradiation field to measure the illuminance on the baby’s body surface.


The case was a 33-week-old gestational boy with a birth weight of 2,028 g who presented with extreme hyperbilirubinemia due to polycythemia. We adopted double-LED phototherapy as a bridging treatment to ET with the informed consent of the patient’s parents. For double-LED phototherapy, neoBLUE (Natus Medical, Pleasanton, CA, USA) with peak emission at a wavelength of 455 nm and BiliLux LED Phototherapy Light System (Drägerwerk, Lübeck, Germany) with peak emission at a wavelength of 470 nm were used together (Figure 1). By irradiating light from directly above the incubator with NeoBlue and irradiating light from diagonally sideways with BiliLux, double-LED phototherapy became possible. Irradiance was measured during the procedure using a BiliBlanket Meter II (Ohmeda medical, Clark, New Jersey, USA). In addition, we measured oxidative stress markers (Diacron-reactive oxygen metabolites) by FREE Carrio Duo (WISMERLL, Tokyo, Japan) using residual serum obtained from this patient before and after the procedure.

**Informed consent:** The patient’s parents have given written informed consent for the use of personal medical data in research and publication.

## 3 Results

This patient developed hyperbilirubinemia (total bilirubin [TB], 10.8 mg/dL) at 30 h after birth (therapeutic threshold of TB, [>10 mg/dL for LP, >14 mg/dL for HP, and >16 mg/dL for ET]), and LP was initiated. Since the TB levels continued to rise to 20.6 mg/dL and exceeded the threshold for ET at 65 h (therapeutic threshold of TB, [>12 mg/dL for LP, >16 mg/dL for HP, and >18 mg/dL for ET]), double-light-emitting diode phototherapy (DL) was started as a bridging treatment to ET. However, due to a rapid decrease of TB levels to 17.1 mg/dL, ET was not required, and HP was initiated at 70 h. Subsequently, the patient’s TB levels began to rise gradually, so DL was adopted again at 93–110 h after birth. Finally, the patient’s bilirubin levels decreased, and phototherapy was terminated 160 h after birth.

**Figure 2:**
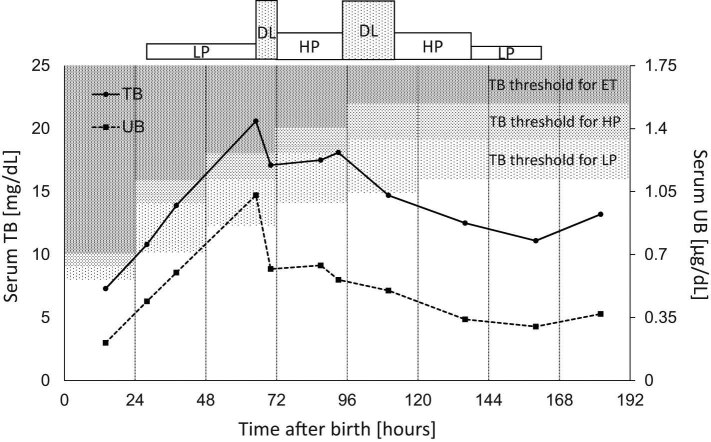
Clinical course of jaundice management. A male infant born at 33 weeks gestation with a birth weight of 2,028 g developed hyperbilirubinemia at 30 h after birth, and low-mode phototherapy (LP) was initiated. Since the total bilirubin (TB) levels continued to rise and exceeded the threshold for ET at 65 h, double-light-emitting diode phototherapy (DL) was started as a bridging treatment to ET. However, due to a rapid decrease of TB levels, ET was not required and high-mode phototherapy (HP) was initiated at 70 h. Subsequently, the patient’s TB levels began to rise gradually, so DL was adopted again at 93–110 h after birth. The patient’s bilirubin levels decreased, and phototherapy was terminated 160 h after birth. The treatment threshold for TB and unbound bilirubin (UB) levels were based on new treatment criteria published by Morioka [1].

**Figure 3:**
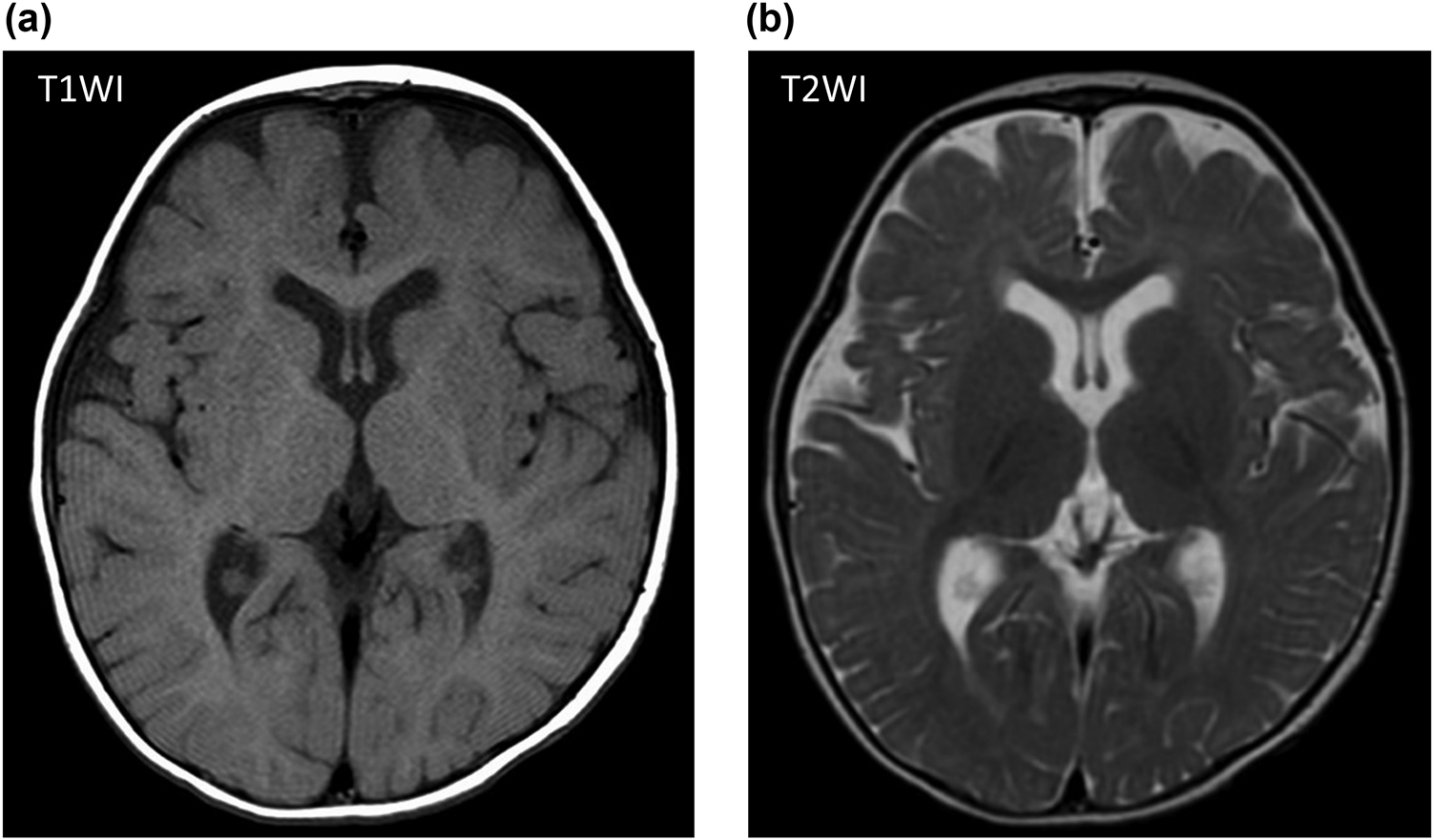
Brain magnetic resonance imaging scans of the patient at 1 year of age. (a) Axial T1-weighted imaging did not show hypointensities in the globus pallidus bilaterally (a sign of kernicterus) and (b) T2-weighted imaging did not show areas of increased signal intensity in the globus pallidus bilaterally (a sign of kernicterus).

In this case, the irradiance of double-LED phototherapy was 85 µW/cm^2^/nm. Intriguingly, the continual increase of bilirubin levels was suppressed immediately after administration of double-LED phototherapy, and ET was not required (Figure 2). No cardiovascular or body temperature disturbances were observed throughout the procedure; no significant increase in oxidative stress, measured as serum Diacron-reactive oxygen metabolites (FREE carrio duo; WISMERLL, Tokyo, Japan) levels, was observed before (*n* = 3; 1.0 ± 0.8 days before treatment, 107 ± 7 Carratelli units) and after (*n* = 4; 1.5 ± 1.1 days after treatment: 107 ± 10 Carratelli units, *p* = 0.97) treatment. In addition, the patient’s neurodevelopment was appropriate for his age at the 1-year follow-up, and no findings of kernicterus, including physical and magnetic resonance imaging findings (Figure 3), were observed.

## 4 Discussion

The principal mechanism of phototherapy for neonatal jaundice is that indirect bilirubin, which is insoluble in water, is converted to water-soluble photoisomers upon irradiation with light at a wavelength of around 480 nm and can be easily excreted in urine or stool [13]. As mentioned earlier, the effect of phototherapy is influenced by several factors; however, it is difficult to alter the wavelength of light irradiance in clinical practice, whereas the irradiance and surface area of the child exposed to light can be easily adjusted by adopting double-LED phototherapy. Intriguingly, increasing the irradiance has been reported to linearly increase the effects of phototherapy [14].

However, the safety issues in the application of double-LED phototherapy remain to be elucidated. Phototherapy using fluorescent tubes is reported to have some adverse effects. DNA damage due to oxidative stress caused by phototherapy has been reported in term infants [15,16]. Due to recent progress in medical technology, LED phototherapy has become widespread for the treatment of neonatal jaundice, and LED phototherapy devices have made it possible to perform phototherapy more safely and easily.

It has been reported that increasing irradiance does not increase DNA damage due to oxidative stress in rats or preterm infants when using an LED phototherapy device [17,18]. Based on these results, it is considered that LEDs may have lesser effects on DNA damage than fluorescent tubes. Given that the goal is to prevent kernicterus, which is a devastating complication of neonatal jaundice, we believe that DL is a well-deserved treatment for neonatal jaundice.

We hypothesized that double-LED phototherapy may be a good treatment strategy to replace ET for infants with severe hyperbilirubinemia; however, further investigations regarding safety issues including acute and long-term complications are needed before clinical adaptation.

## Abbreviations

DL: double-light-emitting diode phototherapy
ET: exchange transfusion
HP: high-mode phototherapy
LED: light-emitting diode
LP: low-mode phototherapy
TB: total bilirubin

## References

[1] Morioka I . Hyperbilirubinemia in preterm infants in Japan: new treatment criteria. Pediatr Int. 2018;60:684–90. 10.1111/ped.13635 29906300

[2] Gutta S , Shenoy J , Kamath SP , Mithra P , Baliga BS , Sarpangala M , et al. Light emitting diode (LED) phototherapy versus conventional phototherapy in neonatal hyperbilirubinemia: a single blinded randomized control trial from coastal India. Biomed Res Int. 2019;2019:6274719. 10.1155/2019/6274719 PMC6487143 31111060

[3] Patra K , Storfer-Isser A , Siner B , Moore J , Hack M . Adverse events associated with neonatal exchange transfusion in the 1990s. J Pediatr. 2004;144:626–31. 10.1016/j.jpeds.2004.01.054 15126997

[4] Jackson JC . Adverse events associated with exchange transfusion in healthy and ill newborns. Pediatrics. 1997;99:E7. 10.1542/peds.99.5.e7 9113964

[5] Steiner LA , Bizzarro MJ , Ehrenkranz RA , Gallagher PG . A decline in the frequency of neonatal exchange transfusions and its effect on exchange-related morbidity and mortality. Pediatrics. 2007;120:27–32. 10.1542/peds.2006-2910 17606558

[6] Maisels MJ . Phototherapy – traditional and nontraditional. J Perinatol. 2001;21(Suppl 1):S93–7. Discussion S104–7. 10.1038/sj.jp.7210642 11803426

[7] Hansen TW . Acute management of extreme neonatal jaundice – the potential benefits of intensified phototherapy and interruption of enterohepatic bilirubin circulation. Acta Paediatr. 1997;86:843–6. 10.1111/j.1651-2227.1997.tb08608.x 9307164

[8] Smitherman H , Stark AR , Bhutani VK . Early recognition of neonatal hyperbilirubinemia and its emergent management. Semin Fetal Neonatal Med. 2006;11:214–24. 10.1016/j.siny.2006.02.002 16603425

[9] Hansen TW . Management of jaundice in newborn nurseries – measuring, predicting and avoiding the sequelae. Acta Paediatr. 2009;98:1866–8. 10.1111/j.1651-2227.2009.01537.x 19821802

[10] Hansen TW . The role of phototherapy in the crash-cart approach to extreme neonatal jaundice. Semin Perinatol. 2011;35:171–4. 10.1053/j.semperi.2011.02.012 21641491

[11] Isenberg JN , Fisch RO . Double-light phototherapy for neonatal hyperbilirubinemia. J Pediatr. 1973;83:116–8. 10.1016/s0022-3476(73)80331-2 4768919

[12] Holtrop PC , Ruedisueli K , Maisels MJ . Double versus single phototherapy in low birth weight newborns. Pediatrics. 1992;90:674–7. 1408537

[13] Okada H , Masuya K , Kurono Y , Nagano K , Okubo K , Yasuda S , et al. Change of bilirubin photoisomers in the urine and serum before and after phototherapy compared with light source. Pediatr Int. 2004;46:640–4. 10.1111/j.1442-200x.2004.01973.x 15660860

[14] Vandborg PK , Hansen BM , Greisen G , Ebbesen F . Dose-response relationship of phototherapy for hyperbilirubinemia. Pediatrics. 2012;130:e352–7. 10.1542/peds.2011-3235 22802603

[15] Tatli MM , Minnet C , Kocyigit A , Karadag A . Phototherapy increases DNA damage in lymphocytes of hyperbilirubinemic neonates. Mutat Res. 2008;654:93–5. 10.1016/j.mrgentox.2007.06.013 18534897

[16] Yahia S , Shabaan AE , Gouida M , El-Ghanam D , Eldegla H , El-Bakary A , et al. Influence of hyperbilirubinemia and phototherapy on markers of genotoxicity and apoptosis in full-term infants. Eur J Pediatr. 2015;174:459–64. 10.1007/s00431-014-2418-z 25209224

[17] van der Schoor LWE , Hulzebos CV , van Faassen MH , Kema IP , de Bruin A , Havinga R , et al. LED-phototherapy does not induce oxidative DNA damage in hyperbilirubinemic Gunn rats. Pediatr Res. 2019;85:1041–7. 10.1038/s41390-019-0367-y 30851724

[18] van der Schoor LWE , van Faassen M , Kema I , Baptist DH , Olthuis AJ , Jonker JW , et al. Blue LED phototherapy in preterm infants: effects on an oxidative marker of DNA damage. Arch Dis Child Fetal Neonatal Ed. 2020;105:628–33. 10.1136/archdischild-2019-317024 32269147

